# An integrated analysis of the effects of maternal broccoli sprouts exposure on transcriptome and methylome in prevention of offspring mammary cancer

**DOI:** 10.1371/journal.pone.0264858

**Published:** 2022-03-09

**Authors:** Itika Arora, Manvi Sharma, Shizhao Li, Michael Crowley, David K. Crossman, Yuanyuan Li, Trygve O. Tollefsbol

**Affiliations:** 1 Department of Biology, University of Alabama at Birmingham, Birmingham, AL, United States of America; 2 Department of Genetics, University of Alabama at Birmingham, Birmingham, AL, United States of America; 3 Department of Obstetrics, Gynecology & Women’s Heath, University of Missouri, Columbia, MO, United States of America; 4 Department of Surgery, University of Missouri, Columbia, MO, United States of America; 5 O’Neal Comprehensive Cancer Center, University of Alabama at Birmingham, Birmingham, AL, United States of America; 6 Integrative Center for Aging Research, University of Alabama at Birmingham, Birmingham, AL, United States of America; 7 Nutrition Obesity Research Center, University of Alabama at Birmingham, Birmingham, AL, United States of America; 8 Comprehensive Diabetes Center, University of Alabama at Birmingham, Birmingham, AL, United States of America; University of Southern California, UNITED STATES

## Abstract

Broccoli sprouts (BSp), a cruciferous vegetable, has shown promising effects on prevention of many types of cancer including breast cancer (BC). BC has a developmental foundation, and maternal nutrition status may influence an offspring’s risk to BC later in life. What is less understood, however, is the influence of maternal nutrition intervention on reversing epigenomic abnormalities that are essential in BC programming during early development. Our research focused on how maternal exposure to BSp diet prevents offspring BC and investigation of possible epigenetic mechanisms during these processes. Our results showed that maternal feeding of BSp can prevent mammary tumor development in the offspring of a transgenic mouse model. Through comprehensive integrated multi-omics studies on transcriptomic and methylomic analysis, we identified numerous target genes exhibiting significantly differential gene expression and DNA methylation patterns in the offspring mammary tumor. We discovered that maternal exposure to BSp diet can induce both gene and methylation changes in several key genes such as *Avpr2*, *Cyp4a12b*, *Dpp6*, *Gria2*, *Pcdh9* and *Tspan11* that are correlated with pivotal biological functions during carcinogenesis. In addition, we found an impact of maternal BSp treatment on DNA methyltransferase and histone deacetylases activity. Our study provides knowledgeable information regarding how maternal BSp diet influences key tumor-related gene expression and the epigenetic changes using a genome-wide perspective. Additionally, these findings provide mechanistic insights into the effectiveness of maternal BSp administration on the prevention of BC in the offspring later in life, which may lead to an early-life BC chemopreventive strategy that benefits the progenies’ long-term health.

## Introduction

Cancer is the second leading cause of death in the United States [1]. Even though there are over one hundred types of different cancers, malignancies of the breast, lung, prostate and colorectum are the leading causes of higher mortality rates [2,3]. Breast cancer (BC) is a leading cause of cancer among women in the United States, and it has been related to both genetic and environmental factors [4,5]. Individual vulnerability to BC can be adversely affected by environmental factors such as diets and nutrition during the lifetime [6]. Early-life events, such as dietary/nutrition intake during prenatal, gestational and postnatal lactational stages, are believed to have a profound impact on offspring’s health and disease outcomes later in life.

Epigenetic mechanisms such as histone modifications and DNA methylation play a significant role on early development by modulating chromatin structure and accessibility to transcriptional factors. DNA methylation is catalyzed by a group of DNA methyltransferases (DNMTs) that dynamically regulate the methylation state of DNA. The methyl group is relocated from S-adenosyl-L-methionine (SAM) and positioned on the 5-position of cytosine in specific CpG dinucleotides by DNMTs [7]. The individual epigenome is established during early embryogenesis, wherein epigenetic reprogramming mechanisms form distinctive epigenetic landmarks [8]. These distinct epigenetic profiles can be retained throughout the lifespan and may be inherited through generations via germline-mediated epigenetic inheritance, thereby ensuring dependable gene transcription regulatory mechanisms in the offspring [9]. Thus, epigenetics may serve as a mechanistic connection between maternal exposure and early-life disease programing [10–13].

Abnormal epigenetic regulation has been well documented to contribute to cancer development and progression [14–16]. It is also becoming increasingly apparent that dysregulation of epigenetic reprogramming during early embryogenesis is associated with a wide range of developmental and congenital abnormalities as well as phenotypic alterations later in life, such as different susceptibility to certain diseases [17]. As a result, this sensitivity to environmental exposure during early life offers a unique opportunity to analyze epigenetic profiles, which ultimately may lead to favorable consequences such as prevention of certain diseases or disorders in the progenies [18]. A number of maternal diets with potential epigenetic regulatory properties has been shown to influence epigenetic reprogramming processes through transplacental effects [6,18,19]. Sulforaphane (SFN), a bioactive dietary component found in cruciferous vegetables like BSp, is a potent epigenetic regulator and chemopreventive agent against a wide variety of human diseases including BC both *in vitro* and *in vivo* [18,20,21]. Studies have shown that SFN in BSp can exhibit chemopreventive properties through various mechanisms including induction of cell cycle arrest, apoptosis, and activation of phase I Cytochrome P450s (CYP) enzymes and phase 2 detoxifying enzymes [22–24]. SFN in BSp has recently received considerable attention because of its ability to influence epigenetic processes by targeting key epigenetic modulators like histone deacetylases (HDACs) and DNMTs. This could lead to local or global changes in epigenetic hallmarks, eventually influencing gene expression profiles [20,21,25,26]. Our previous studies have demonstrated that SFN can activate the estrogen receptor (*ER*) gene and suppress human telomerase reverse transcriptase (*hTERT*) in ER-negative [ER(-)] BC cells via epigenetic processes [20,21]. Dietary administration of orally-fed BSp in xenograft mouse model can lead to an overall increase in histone acetylation through HDAC inhibition [20,25]. These findings suggest that dietary BSp can inhibit BC by influencing key tumor-related genes via epigenetic machinery. Even though research on SFN and its supplemented BSp diet is very promising for cancer chemoprevention, determination of their effectiveness for cancer prevention through maternal exposure remains a significant challenge. Importantly, our previous studies demonstrated temporal efficacy of BSp in prevention of BC later in life, suggesting consuming SFN from BSp early in life may be more beneficial than consuming SFN later in life for reducing BC [11,27].

In this study, we hypothesized that a BSp bioactive diet may influence early development by modulating epigenetic profiles, thereby resulting in altered susceptibility to BC in the offspring. We investigated the maternal BSp impact on offspring mammary tumor development in a transgenic mouse model, C3(1)-SV40 Tag (SV40), through epigenetic mechanisms. Our integrative framework of reduced-representation bisulfite sequencing (RRBS) for methylome analysis and RNA-sequencing (RNA-seq) for transcriptome analysis helped to assess whether dietary feeding of maternal BSp is effective in neutralizing epigenomic aberrations in key tumor-related genes, which can lead to inhibition of BC formation and progression in the offspring. Our findings demonstrated that maternal BSp feeding can not only induce global DNA methylomic changes, but also affect key regulatory gene expression in the offspring of transgenic mice, which may contribute maternal BSp-induced chemopreventive effects against BC later in life. Overall, this study provides an in-depth understanding of the preventive effects of maternal administration of BSp on offspring BC, which may potentially facilitate development of a novel maternal dietary intervention strategy to reduce BC incidence for the future generations.

## Materials and methods

### Animals

A transgenic mouse model, C3(1)-SV40 TAg (FVB-Tg(C3-1-TAg)cJeg/JegJ) (SV40), was used in this study. Animals were originally purchased from the Jackson Laboratory and further colonized in our laboratory. Female SV40 mice develop ER(-) mammary tumors caused by an overexpressed transgene at early age with a median tumor latency of ~15 wks [28]. The dams were bred at 10–12 wks age to obtain sufficient colonies for future investigations. Offspring mice were weaned and genotyped at 4 wks of age. All mice were housed in the University of Alabama at Birmingham’s Animal Resource Facility, kept in a 12-hour light/dark cycle, 24 ± 2°C temperatures, and 50±10% humidity. All animals had free access to food and water. The animal study was reviewed and approved by the Institutional Animal Use and Care Committee of the University of Alabama at Birmingham (IACUC; Animal Project Numbers: 10088, 20653 and 20671).

### Dietary treatment

**Fig 1:**
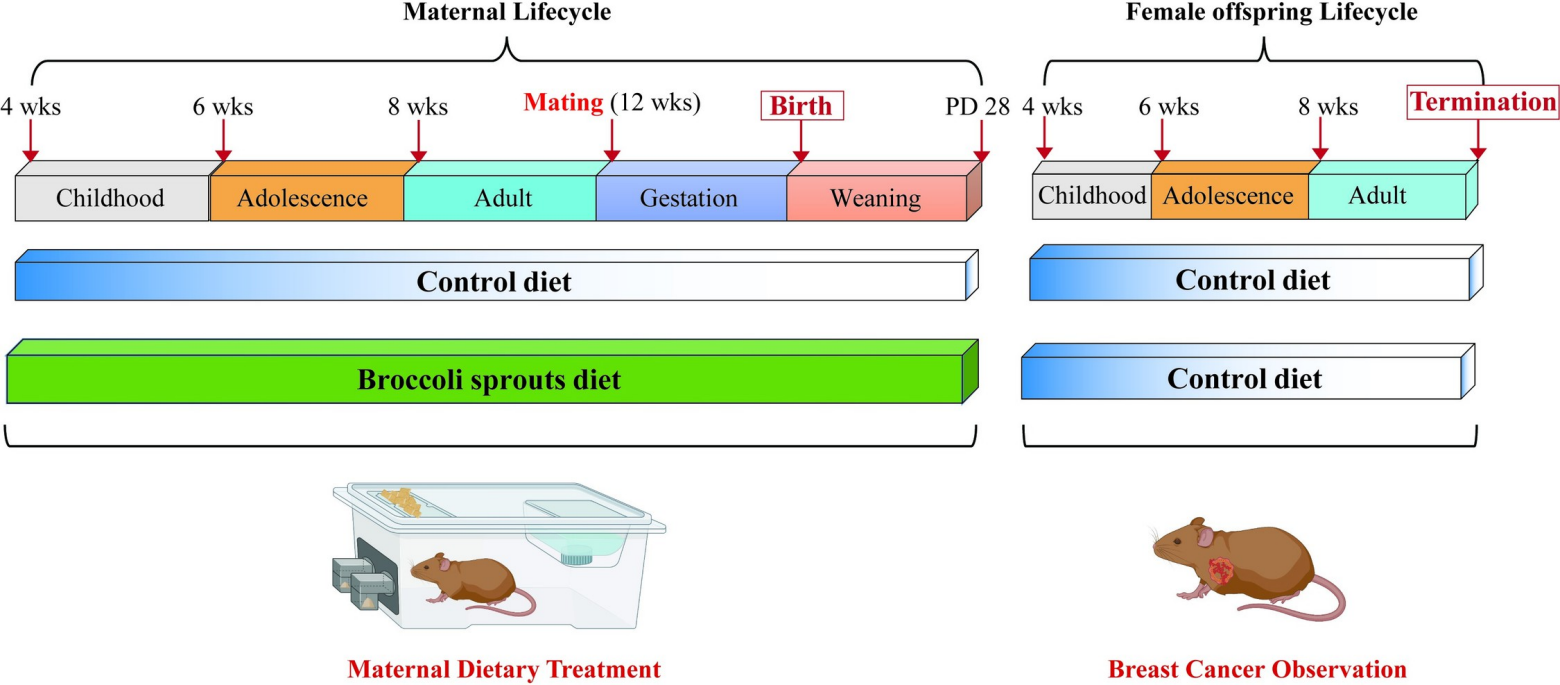
Schematic representation of study design. The upper bar represents different life stages in the mother and female offspring. Transgenic mice were administered either Control or BSp diet resulting in 2 treatment groups (10 mice/group): 1) Control: mothers were fed ad libitum (AL) with the Control AIN-93G diet; 2) Maternal BSp treatment (BSp): The BSp diet was given to the mothers from early life at 4 wks of age until weaning at postnatal days (PD28), representing a persistent eating habit throughout the lifespan. Mothers were mated around 12 wks of age following the period of 3 wks of gestation, resulting in ~20 offspring mice in each group. Offspring mice were weaned at 4 wks of age and maintained on Control diet throughout their lifespan.

Dams (10 mice/group) were administered either Control AIN-93G diet or customized BSp chow diet from TestDiet (St. Louis, MO) from 4 wks of age as demonstrated in Fig 1. The modified AIN-93G was comprised of 26% (w/w) BSp, which was obtained from Natural Sprout Company (Springfield, MO). The formulation of BSp diet has been demonstrated to be safe with no adverse effects on either the mother or offspring from our previous studies [11,27]. Animal diets were stored in an airtight container and away from light in 4°C refrigeration for up to 6 months or stored at -20°C for later use. Mice were mated at 12 wks of age and male mice were separated from female mice after conception. The female offspring mice were weaned at 3 wks of age and administered the Control diet throughout their lifespan.

### Tumor collection and evaluation

We observed tumor growth in female offspring mice. We utilized tumor latency as the primary outcome followed by tumor weight due to the spontaneous formation of mammary tumors in the transgenic mouse model. Tumor incidence was determined on a weekly basis [11,27]. Mice were euthanized and the experiment was terminated upon the Control mice reaching a 100% tumor rate or when the mean tumor diameter of the individual animal exceeds 1.0 cm. The breast tumors were collected, weighed, and used for additional analysis upon the termination of the experiment.

### RNA sequencing (RNA-seq) analysis

In this study, we used 3 biological and 4 technical replicates in the control group; and 4 biological and 4 technical replicates in the BSp groups. Total RNAs were extracted from mammary tumors from Control and maternal BSp treatment groups (3–4 mice/group) using TRIzol reagent (Sigma-Aldrich, St. Louis, MO) followed by the standard protocol. The RNA concentrations were quantified using a NanoDrop spectrophotometer, and the integrity of the RNA was assessed using an RNA Nano bioanalyzer chip. Further, RNA samples with integrity number more than 7 were retained for sequencing. To generate sequencing libraries, RNAs were reverse transcribed using Agilent SureSelect Strand Specific mRNA library kit (Agilent, Sant Clara, CA). At the UAB Genomics Core Laboratories, RNA-seq pair-end libraries were sequenced on an Illumina NextSeq500 (Illumina Inc., San Diego, CA). For each mRNA preparation, around 25~30 million sequencing reads were generated and processed for quality assessment using fastQC to examine the quality of the raw fastq data per sample. RNA sequences were aligned to the UCSC Genome Browser mouse GRCm39/mm10 reference sequence (UC, Satan Cruz) using Kallisto (v0.43.1-intel-2017a) generating thereby a transcription-level estimates (or abundance estimate) file for each sample/treatment group [29]. Additionally, the tximport package in R (v1.20.1) was used to import a transcript-level estimated file, and the transcript-level information was summarized to gene-level with their corresponding expression levels [30].

### Differentially expressed gene (DEGs) analysis

To examine the impact of maternal BSp on transcriptional alterations in mammary tumors of SV40 mice offspring, we used the R/Bioconductor limma (v3.48.3) package in platform to assess DEGs via fold change (FC) estimates and quantile normalization, which is a method of attempting to match gene count distributions across samples in a dataset. The criterion for differential expression between the Control and maternal BSp treatment groups was set at a false discovery rate (FDR) of 0.05. The average log2 expression values for the Control and maternal BSp treatment groups were computed using normalized DESeq counts for each sample. The difference in average log2 expression values between the Control and maternal BSp treatment groups was used to compute the ratio values or FC for each gene, wherein the positive value for a transcript is symbolized as upregulation and negative value for a transcript as down-regulation.

### Reduced representation bisulfite sequencing (RRBS) analysis

Genomic DNAs were extracted from the mammary tumors of offspring mice using DNeasy Blood and Tissue Kit (Qiagen, Germantown, MD). DNA concentration was determined using a NanoDrop spectrophotometer and the DNA quantity was measured using Qubit. DNA samples from Control and maternal BSp treatment groups (3–4 mice/group) were used for RRBS analysis.

To minimize biological variance, an identical set of biological samples was utilized for both RRBS and RNA-Seq analysis. RRBS was carried out at the UAB Genomics Core Laboratories, according to a standard manufacturer’s protocol [31]. Bisulfite-converted DNA libraries were constructed, amplified, and single-end reads were sequenced on an Illumina NextSeq 500 platform. All the samples yielded an average of 50 million high-quality 50-bp paired end reads, indicating that bisulfite conversion efficiency was >98%. Furthermore, FastQC (http://www.bioinformatics.babraham.ac.uk/projects/fastqc/) was used to evaluate the quality of RRBS raw reads. Trim Galore (http://www.bioinformatics.babraham.ac.uk/projects/trim galore/) was further employed in order to perform adaptor trimming followed by the removal of trimmed reads which were shorter than 20 bp at the end of sequences based on NuGEN Ovation RRBS system. Trimmed bisulfite reads were mapped to the UCSC Genome Browser mouse GRCm38/mm10 reference sequence using the Bismark aligner with default parameter settings [32]. Furthermore, methylation locus analysis was extracted using a post alignment and Bismark additional methylation extractor script that runs on Bismark result files, thereby generating Bismark coverage output dataset which was further employed for downstream statistical analysis.

### Identification of differentially methylated loci (DML) and differentially methylated regions (DMRs)

Statistical tests were performed at each CpG site to identify DML and DMRs to detect differential methylation status between the Control and maternal BSp treatment groups by using methylKit package in R (v4.1.1). The methylation files generated using Bismark were used an input to generate CpG region profiles. Subsequently, methylKit [33] was used to identify Differentially methylated genes (DMGs) with DMR calling threshold being set to < 0.1. Additionally, a sliding linear model with a p-value <0.05 was used to define differential methylation. A stringent statistical test, DSS (Dispersion Shrinkage for Sequencing Data) [34], was also used for differential methylation analysis due to the biological diversity among the replicates and the variance in sequencing depth.

### Integrated analysis of RNA-seq and RRBS

We integrated transcriptomic and methylomic data by merging the major DEGs and DMGs using the ggplot2 package (v3.3.5) in R (v4.1.1) to explore the potential association between gene transcription and DNA methylation. To further visualize the correlation between DEGs-DMGs, a scatter plot with log_2_FC and methylation difference was generated using ggplot2 package (v3.3.5) in R (v4.1.1). Subsequently, a heatmap between Differentially methylated (DM) and DE in response to maternal BSp treatment group was generated using ComplextHeatmap package (v2.8.0) in R (v4.1.1) to visualize the transcription and methylation level changes between these overlapping genes.

### Quantitative real-time PCR

The QIAGEN RNeasy Plus Kit was used to extract total RNA from offspring mammary tumors of Control and maternal BSp treatment groups. Following RNA extraction, the iScript cDNA synthesis kit (BioRad, Hercules, CA, Biorad) was used to reverse transcribe the RNA to cDNA according to the manufacturer’s instructions. Specific primers for the target genes of interest were synthesized by Integrated DNA Technologies (Coralville, Iowa). Gene expression for the selected target genes was performed in triplicate and further analyzed using real-time PCR by SsoAdvanced Universal SYBR GreenER qPCR Supermix (BioRad) in BioRad CFX Connect Real-time System. These selected target genes were *Avpr2*, *Cyp4a12b*, *Dpp6*, *Gria2*, *Pcdh9* and *Tspan11* and *β-actin* served as an internal Control. We used 1 μL of cDNA, 5 μL of Bio-iTaq Rad’s SYBR green, 2 μL of nuclease-free water, and 1 μL of forward and reverse primers for specific genes of interest in a total volume of 10 μL for the PCR experimental setup. Thermal cycling was performed for 4 mins at 94°C, followed by 35 PCR cycles (94°C for 15 secs, 60°C for 30 secs, and 72°C for 30 secs). The primer sequences were used as following: 5′-GTGGACAGTGAGGCCAGGAT-3′ (F) and 5′-GATTACTGCTCTGGCTCCTAGCA-3′ (R) for *β-actin*; 5′-CCAAGACCGTGAAGAGGATGAC-3′(F) and 5′-GAGACACTGCTACTGAAGGAA-3′ (R) for *Avpr2*; 5′-GTGTCCTCTAATGGCTGCTT-3′ (F) and 5′-TTTCCAACTCTTCCTCATCCTG-3′ (R) for *Cyp4a12b*; 5′-GTGACCAAGATCCTGTCCTATG-3′ (F) and 5′-CACTGCCTGTTGAAGTCATCT-3′ (R) for *Dpp6*; 5′-CACTTCGGAGTTCAGACTGAC-3′ (F) and 5′-AATCGCATAGACGCCTCTTG-3′ (R) for *Gria2*; 5′-CCACTAGTTCAGATCACTTCAGT-3′ (F) and 5′-CTGGTCGTAGAATTCGTCCTG-3′ (R) for *Pcdh9*, and 5′-CCACCCCTCCAACATCTAC-3′ (F) and 5′-TGAGACCATCCCACAGAT-3′ (R) for *Tspan11* gene. The mRNA abundance for the genes of interest was quantified using ΔCT method and relatively expressed to β-actin mRNA. Data were depicted as means ± standard error of means and Student’s t test was used to make statistical comparisons with a *p* value of less than 0.05.

### DNMT and HDAC activity assay

DNA was extracted from the harvested mammary tumors using the EpiQuik Nuclear Extraction Kit (EpiGentek) based on the manufacturer’s protocol. Further, nuclear protein was extracted for determination of overall DNMT and HDAC enzymatic activities using the EpiQuik DNMT Activity/Inhibition Assay Ultra Kit (EpiGentek) and EpiQuik HDAC Activity/Inhibition Assay Kit (EpiGentek), respectively.

### Statistical analysis

Statistical significance between the values of the Control and maternal BSp treatment groups was analyzed using one-way ANOVA followed by Tukey’s test for multiple comparisons using GraphPad Prism 8.00 version, aside from RNA-seq and RRBS integrative studies. Tumor incidence and significance were determined using Chi-square test and Fisher’s exact test. Values were presented as mean ± SD (standard deviation) and statistically significant outcomes were represented as ** for *p* value < 0.01 and * for *p* value < 0.05.

### Gene set functional enrichment analysis

For downstream analysis, target transcripts at the transcriptomic and methylomic levels (*p*-value <0.05) were employed to perform Gene Ontology (GO) enrichment. We used a web-based tool, WEB-based Gene SeT AnaLysis Toolkit (WebGestalt) to perform an over-representation analysis to identify keys biological processes, molecular functions and cellular components with a significance level of 5% FDR [35].

## Results

### Maternal BSp treatment resulted in preventive effects on mammary tumor development in transgenic offspring mice

**Fig 2:**
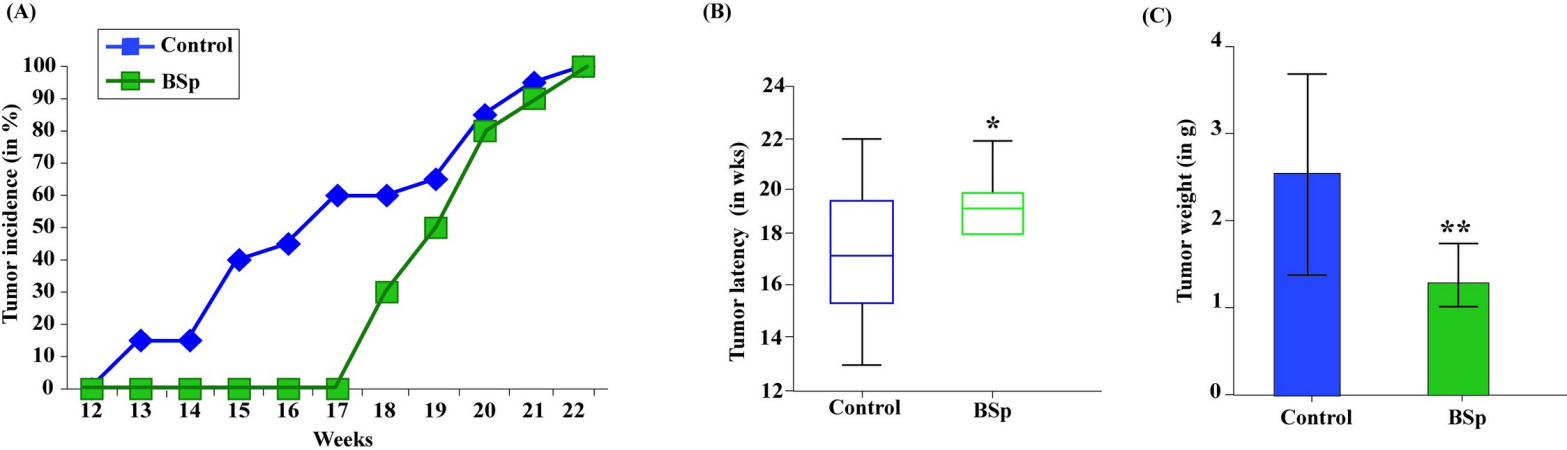
Tumor growth in female progenies with maternal dietary administration of BSp. SV40 dams were administered with regular Control diet or customized 26% BSp diet throughout the lifespan. Female offspring mice were weaned at 4 wks of age and maintained on Control diet and monitored for mammary tumor development. **(A)** Tumor incidence measured by percentage over the whole population. **(B)** Median tumor latency in the offspring. **(C)** Average tumor weight among the dietary treatment group. Columns, mean; bars, standard deviation; *p value < 0.05, **p value < 0.01, significantly different from the Control group.

We assessed the impact of maternal feeding of BSp diet on mammary tumor development in the offspring of SV40 transgenic mice that form spontaneous ER(-) mammary tumors caused by overexpression of the oncogene. Although carcinogenic amplicons or activation do not represent all breast tumorigenesis, the molecular cascades and subsequent signaling inductions elicited by these activated oncogenes are analogous to underlying molecular events during human BC tumorigenesis [28]. The concentration of BSp in the diet (26% w/w) utilized in this investigation is equal to daily BSp intakes of ~234 g for an adult human, indicating that this approach is pharmacologically feasible and has translational potential [36]. As a result, we found that maternal BSp treatment exhibited inhibitory effects on tumor growth in offspring mice compared to the Control, especially between the period of 12 to 17 wks (Fig 2A). Statistically, maternal BSp group significantly extended 11.8% tumor latency compared to the Control (Fig 2B). Furthermore, tumor weight was significantly decreased in the offspring by maternal BSp treatment with *p* value < 0.001 (Fig 2C). Overall, maternal feeding of BSp was effective in inhibiting mammary tumor development in offspring mice.

### Maternal dietary administration of BSp led to genome-wide alteration in transcriptome of SV40 mice offspring

According to our previous publications, maternal exposure to BSp diet has shown substantial protective effects against mammary cancer in the offspring of different transgenic mouse models [11]. To better understand the underlying biological mechanisms, we evaluated global gene transcriptional level in the tumors of female offspring of SV40 transgenic mice to identify differentially expressed (DE) transcripts in response to maternal BSp treatment that may link its effects to early BC prevention. Upon mammary tumor observation in the offspring, the mammary tumors were harvested at termination of the experiment (N_Control_  = 4, N_BSp_  =  3) and used for further RNA-seq analysis (Fig 1). The RNA-seq data were first normalized for linear modeling, and the outliers were found by creating a boxplot across all samples in each treatment group (S1 Fig). To detect gene expression alterations, the distribution of samples was analyzed using a histogram (S2 Fig). Subsequently unsupervised principal component analysis (PCoA) was applied on gene expression profiles for individual samples among different treatment groups (S3 Fig).

From a total of 11,576 DE transcripts (genes) detected (S1 File), we identified 210 transcripts that are of special interest with log_2_ fold change above 2 along with a strict statistical analysis of FDR<0.05. Amongst them, 74 genes were upregulated and 136 were down-regulated. The list of the 210 DE transcripts is elucidated in S2 File. Additionally, Tables 1 and 2 exhibit the top 20 upregulated and down-regulated identified target genes in comparison with Control and BSp treatment, ranked by the statistical significance (log_2_FC).

**Table 1:** Top 20 differentially upregulated target genes in the offspring with the maternal fed BSp diet (ranked by the statistical significance).

Gene	Gene expression fold-change (log_2_FC) (BSp vs Control)	Average differential expression (BSp vs Control)	p value for differential expression (BSp vs Control)	False discovery rate (FDR)
*Lhx1*	7.01	0.77	5.15E-05	2.96E-03
*Foxn1*	5.68	0.80	3.10E-06	7.85E-04
*Kif6*	5.62	-1.95	1.07E-04	4.33E-03
*Slc6a7*	5.51	3.05	6.79E-08	2.17E-04
*Gm14322*	4.86	-3.18	1.24E-06	5.10E-04
*Gm14440*	4.71	-0.03	1.76E-04	5.84E-03
*Crispld1*	4.70	0.31	6.63E-07	3.92E-04
*Gm20547*	4.49	-4.27	7.91E-06	1.28E-03
*Hba-a2*	4.40	1.88	1.46E-07	2.17E-04
*Gm6465*	4.24	-4.49	1.08E-05	1.38E-03
*Ano4*	4.09	1.85	5.22E-06	9.61E-04
*Prkcq*	4.09	-2.93	4.41E-05	2.71E-03
*Brinp2*	3.98	-3.64	1.84E-05	1.76E-03
*Fam25c*	3.90	-1.75	1.58E-04	5.44E-03
*Sectm1b*	3.87	3.48	1.02E-06	4.59E-04
*Prl2c2*	3.85	-0.95	4.36E-04	9.59E-03
*Clec4e*	3.85	-0.41	9.78E-07	4.59E-04
*Pcdha11*	3.76	-4.65	4.00E-05	2.54E-03
*Cstdc4*	3.76	-4.06	1.74E-05	1.73E-03
*Cfap47*	3.74	-3.53	1.95E-05	1.77E-03

**Table 2:** Top 20 differentially down-regulated target genes in the offspring with maternal BSp treatment (ranked by the statistical significance).

Gene	Gene expression fold-change (log_2_FC) (BSp vs Control)	Average differential expression (BSp vs Control)	p value for differential expression (BSp vs Control)	False discovery rate (FDR)
*Slx4ip*	-6.85	-1.72	1.28E-07	2.17E-04
*Gm20431*	-6.58	-1.77	1.78E-07	2.39E-04
*Utf1*	-6.12	-3.52	7.86E-06	1.28E-03
*Mcf2*	-6.09	-3.98	5.06E-08	2.17E-04
*Wbp2nl*	-5.93	-1.79	4.92E-05	2.91E-03
*Sytl3*	-5.36	-3.61	4.07E-06	9.26E-04
*Fmn2*	-5.33	-1.19	1.03E-06	4.59E-04
*Col26a1*	-5.29	-3.92	2.04E-06	6.55E-04
*Adamtsl5*	-5.29	-3.55	1.45E-07	2.17E-04
*Gm14288*	-5.13	-4.05	1.11E-05	1.40E-03
*Gjb1*	-5.05	-0.72	4.69E-06	9.31E-04
*Cdh10*	-4.85	-4.37	2.53E-07	2.79E-04
*Gm49353*	-4.84	-4.17	2.36E-06	6.61E-04
*Gm10340*	-4.82	-4.33	8.88E-08	2.17E-04
*Kcnh8*	-4.78	-3.40	1.03E-05	1.35E-03
*Zfp982*	-4.73	-2.10	1.96E-06	6.55E-04
*Scn9a*	-4.53	-4.49	3.92E-07	3.29E-04
*Tenm3*	-3.96	-0.88	7.62E-05	3.62E-03
*Trnp1*	-3.81	-3.87	9.31E-06	1.35E-03
*Ccdc160*	-3.76	-4.36	1.79E-05	1.75E-03

**Fig 3:**
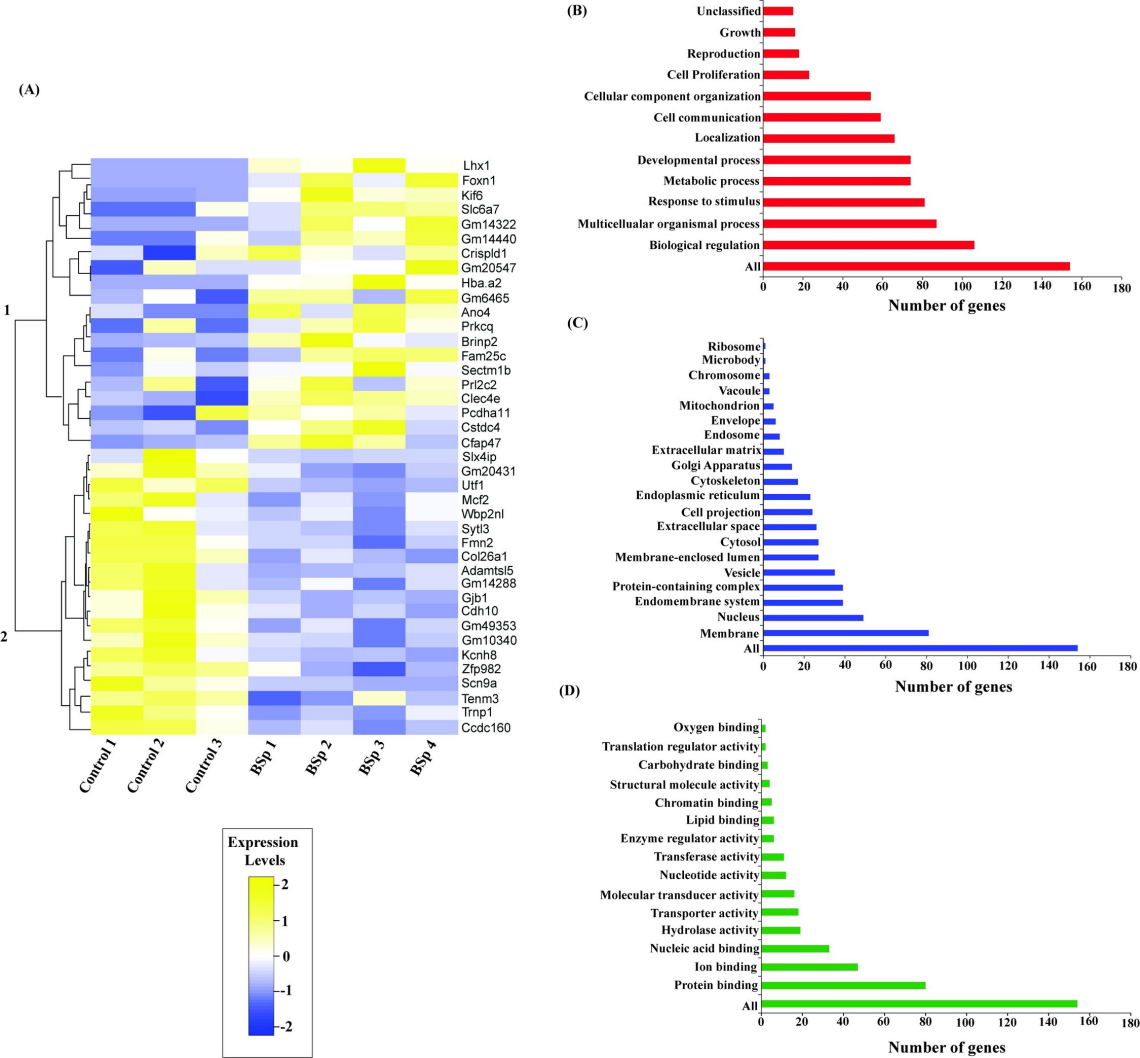
Maternal dietary administration of BSp influenced offspring transcriptome. **(A)** Heatmap representing top 20 upregulated and down-regulated target genes between the Control- and maternal fed BSp-born offspring mice based on *q* value. Each row corresponds to DE transcripts and each column represents biological replicates in Control (N  =  3) and the BSp (N  =  4) treatment groups. Blue color denotes lower expression levels and red color denotes higher expression levels. **B-D,** GO term analysis of DE transcripts for the maternal BSp treatment. Bar graph of GO enrichment analysis for DE transcripts in **(B)** Biological processes, **(C)** Cellular components and **(D)** Molecular functions.

To better visualize the transcriptional level changes induced by maternal feeding of BSp diet in mice offspring, we generated a heatmap with the top 20 upregulated and down-regulated target genes between rows representing the DE transcripts and columns representing biological replicates in the Control and BSp treatment groups (Fig 3A).

Additionally, we used WebGestalt software to perform GO SLIM subset analyses, which identified key clusters within diverse biological functions to acquire a comprehensive knowledge of DEGs during tumor development and oncogenic transition. As shown in Fig 3B, in the maternal BSp treatment group, among the biological processes category, the major subsets were “biological regulation” and “multicellular organismal process” and “response to stimulus”. Similarly, in the cellular component category, the most abundant terms were “membrane’, “nucleus” and “endomembrane system” (Fig 3C). In the molecular function category, the most frequent terms were related to “protein binding”, “ion binding” and “nucleic acid binding” (Fig 3D). These results indicate that maternal dietary administration of BSp may play a pivotal role in the regulation of specific regulatory pathways, which might potentially demonstrate its induced transplacental effects on BC prevention in the offspring.

### Dietary administration of maternal BSp altered genome-wide DNA methylation profiles in the offspring mammary tumors

Considering the significant influence of maternal nutrition on epigenetic reprogramming processes during early development, we used RRBS analysis to test the impacts of maternal fed BSp diet on genome-wide DNA methylation profile changes that may contribute to the outcome of breast cancer later in life. A total of seven libraries was generated for Control and maternal BSp groups, which were further aligned to the *Mus musculus* reference genome (GRCm38/mm10) by Bismark. Furthermore, we defined DMRs and DMLs among the Control and BSp group with a minimum range of 500 bp. Additionally, we performed dependency correction for DMRs to further identify statistically significant differentially methylated genes in response to maternal BSp intervention.

**Fig 4:**
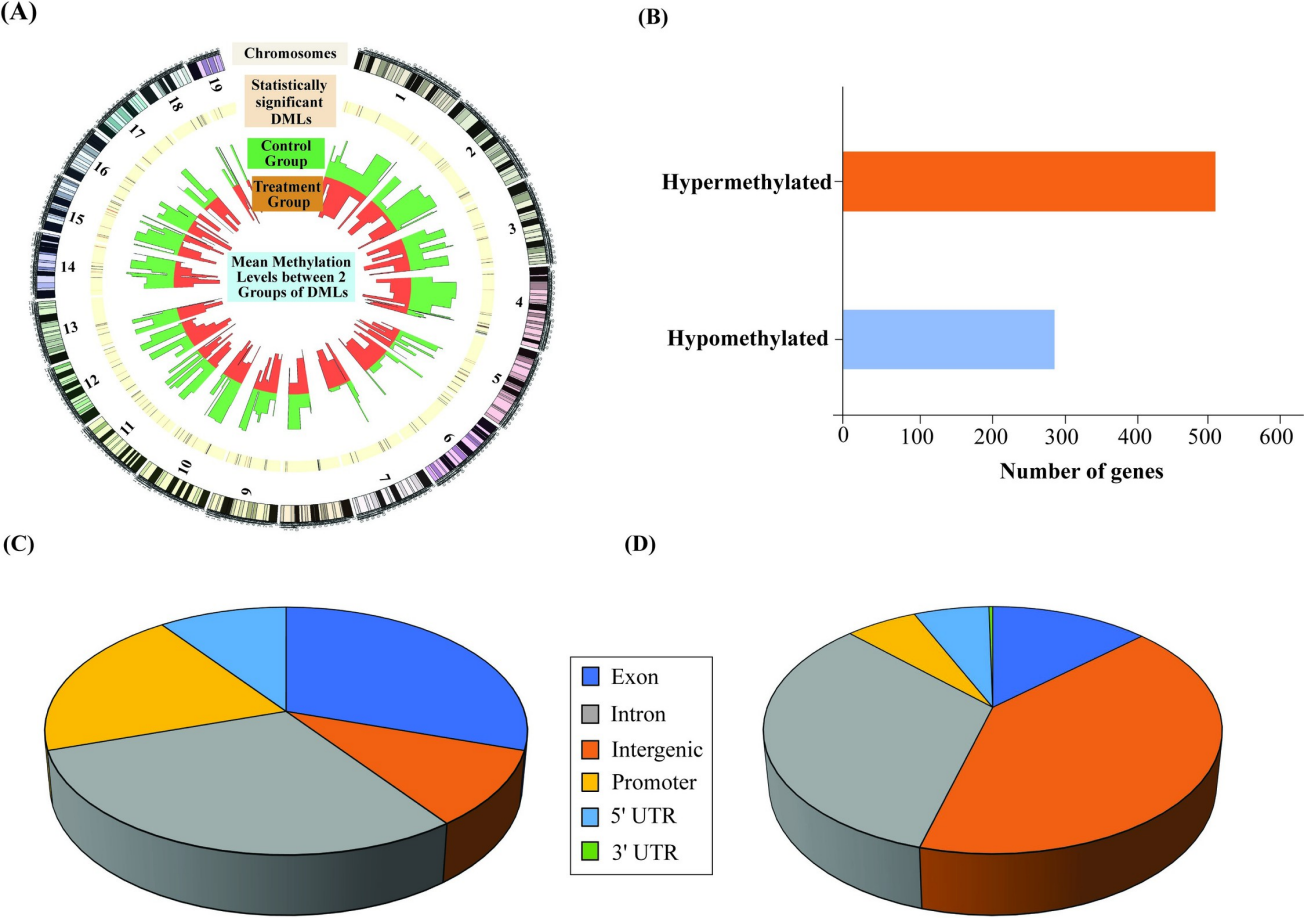
Maternal dietary administration of BSp influenced offspring methylome. RRBS analysis was used to determine genome-wide DNA methylation profiling in Control- and maternal BSp-treated SV40 offspring mammary tumors. **(A)** Genome-wide distribution of DML. Bands from the outside to inside represent chromosomes (grey), significant DML (yellow) distributed between Control (green) and maternal BSp treatment (orange) in the offspring mammary tumors of SV40 transgenic mice. **(B)** Bar plot representing differentially hypomethylated or hypermethylated DMR in response to maternal BSp treatment. The height in the bar plot represents the total number of DMGs. **(C and D)** Pie charts represent genomic distribution of DMRs **(C**) and DMLs **(D)** in response to maternal BSp treatment. Grey color represents intronic region, dark blue represents exonic region, orange represents intergenic region, yellow represents promoter region, light blue represents 5’ untranslated regions (5’ UTR) and green represents 3’ untranslated regions (3’ UTR).

Out of 2890 DMLs, 1837 loci (63.56%) were hypomethylated and 1053 loci (36.44%) were hypermethylated in response to maternal BSp administration (S3 File), which were illustrated to distribute across the genomic and chromosomal regions (Fig 4A). As a result, we identified 803 DMRs amongst which 291 DMR (36.24%) were hypomethylated, while 512 DMR (63.76%) were hypermethylated (Fig 4B and S4 File). The list of the all the transcripts (687 genes) that were DM is provided in S5 File. Upon assessing the location distribution of DMRs or DMLs throughout the genome, we found that DMRs were mainly distributed in exonic and intronic regions (Fig 4C) and DMLs were mainly distributed in intronic and intergenic regions (Fig 4D).

### Integrated analysis identified key transcripts with DM due to maternal dietary administration of BSp

We performed integrative analyses by incorporating multi-omics data including transcriptomic and methylomic data to identify DEGs with DM alterations and to investigate potential associations between DNA methylation and transcriptional regulation through maternal BSp treatment-induced BC preventive outcome. Overall, 210 DEGs and 687 DMGs were identified from RNA-seq and RRBS, respectively, amongst which we eventually identified 11 target genes exhibiting both DE (with an average expression of normalized read counts in the abundance of various genes within a transcriptome between the Control-fed and BSp-fed diets) and DM changes. **Table 3** provides a comprehensive depiction of identified target genes with both DE and DM.

**Table 3:** Identified target genes with significant DE and DM changes due to maternal dietary administration of BSp in the offspring of SV40 transgenic mice.

Gene symbol	Gene expression fold-change (log_2_FC) (BSp vs Control)	Average differential expression (BSp vs Control)	p value for differential expression (BSp vs Control)	Chromosomal location	Methylation difference (BSp vs Control)
*Avpr2*	2.09	0.07	1.44E-24	X	-58.56
*Brinp2*	3.98	-3.64	6.42E-06	1	31.86
*Cyp3a41a*	2.59	-2.97	3.59E-16	5	30.73
*Cyp4a12b*	-2.38	-4.71	9.87E-05	4	26.23
*Dcc*	-2.85	-3.99	3.74E-04	18	-35.87
*Dpp6*	-2.84	-1.35	1.13E-06	5	26.80
*Gria2*	-2.98	-4.26	2.37E-05	3	25.41
*Pcdh9*	-2.09	-4.44	1.40E-06	14	28.05
*Six1*	2.37	2.74	5.57E-12	12	53.99
*Tmem213*	-2.60	-4.78	9.95E-09	6	-31.05
*Tspan11*	-3.11	0.57	3.52E-10	6	54.36

**Fig 5:**
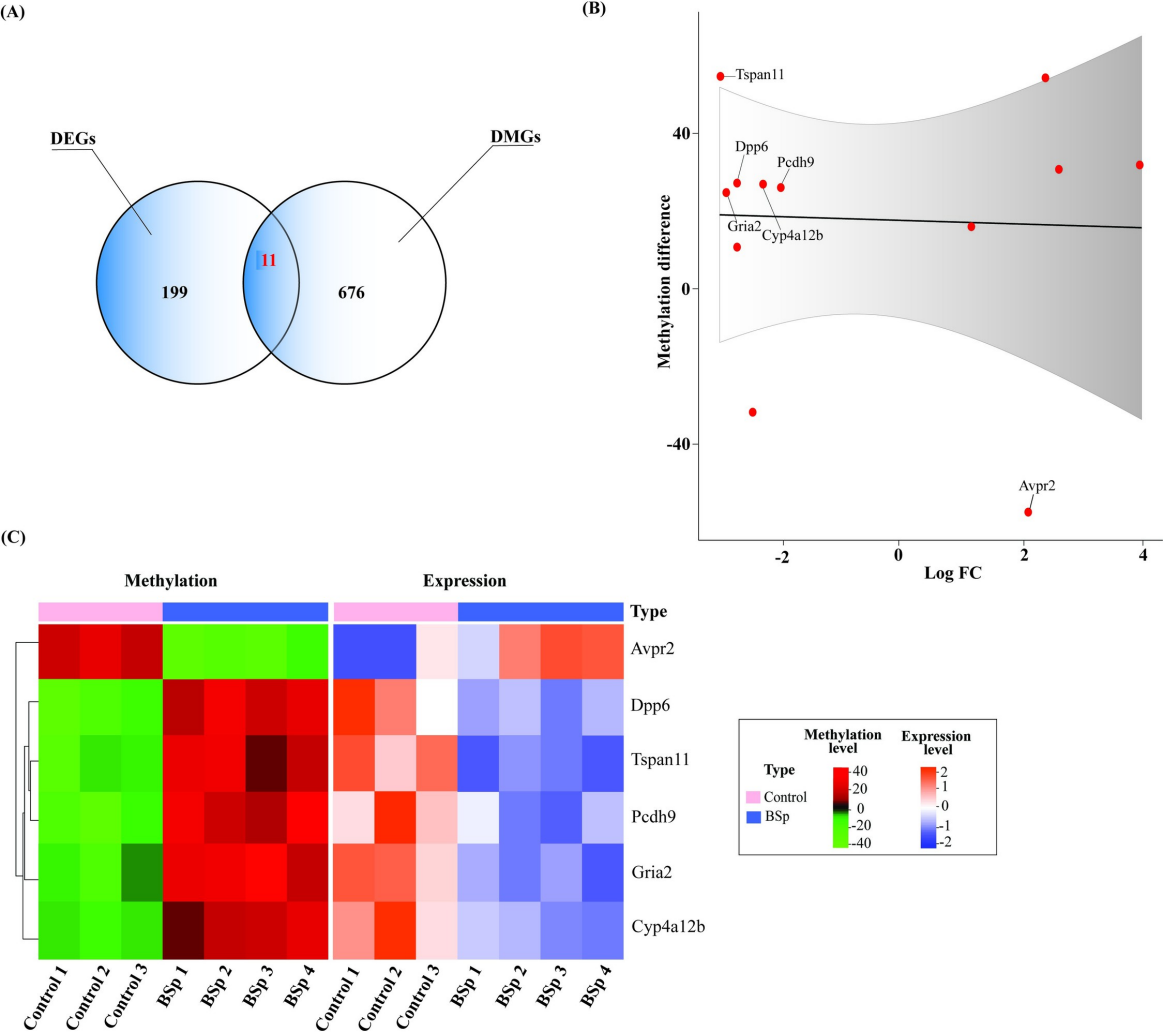
Integration analysis of RNA-Seq and RRBS to identify target genes with both DE and DM. Correlation of DEGs and DMRs by maternal BSp treatment. **(A)** Venn diagram represents 11 unique and overlapped DEGs and DMRs in response to BSp maternal treatment. **(B)** Scatter plot of 11 differentially expressed and methylated genes (dots in red) is represented on the y-axis, while log_10_FC is represented on the x-axis. The gene names of 6 genes showing consistent regulation pattern were labeled. One gene (*Avpr2*) was upregulated and hypomethylated, while five transcripts, *Cyp4a12b*, *Dpp6*, *Gria2*, *Pcdh9 and Tspan11* were down-regulated and hypermethylated. **(C)** Heatmap represents 6 target genes showing both DE and DM in response to maternal BSp treatment based on *p* value. Each row corresponds to DE or DM transcripts and each column represents biological replicates in Control (N  =  3) and maternal BSp (N  =  4) treatment group.

As similar as reported in Table 3, multiple studies with gene expression do not always correlate with the canonical dogma of increased methylation with decreased gene expression and vice-versa. One of the classic exceptions is *TERT* to this rule due to impeding the binding of its many repressors. There are often site-specificity considerations of DNA methylation affecting gene expression for many genes. For instance, a study by Lee et al. in 2019 reported DNA hypermethylation and upregulation of *TERT* expression in different types of cancer [37]. Another study demonstrated DNA hypermethylation by downregulating *TERT* in *Helicobacter pylori*-induced chronic inflammation [38]. Similarly, out of 11 genes showing both DM and DE, *Avpr2* was upregulated and hypomethylated, and 5 genes including *Cyp4a12b*, *Dpp6*, *Gria2*, *Pcdh9* and *Tspan11* were down-regulated and hypermethylated (Fig 5A), indicting consistent regulation pattern between gene transcription and DNA methylation (loss methylation leads to upregulation and vice versa). To better understand the relationship between these 6 transcripts that were showing both DM and DE, we generated a scatter plot between methylation difference and log_10_FC (Fig 5B) and heatmap wherein rows represent DM (left) or DE (right) and columns represents biological replicates for Control and BSp groups (Fig 5C).

### Validation analysis of identified target gene expression

**Fig 6:**
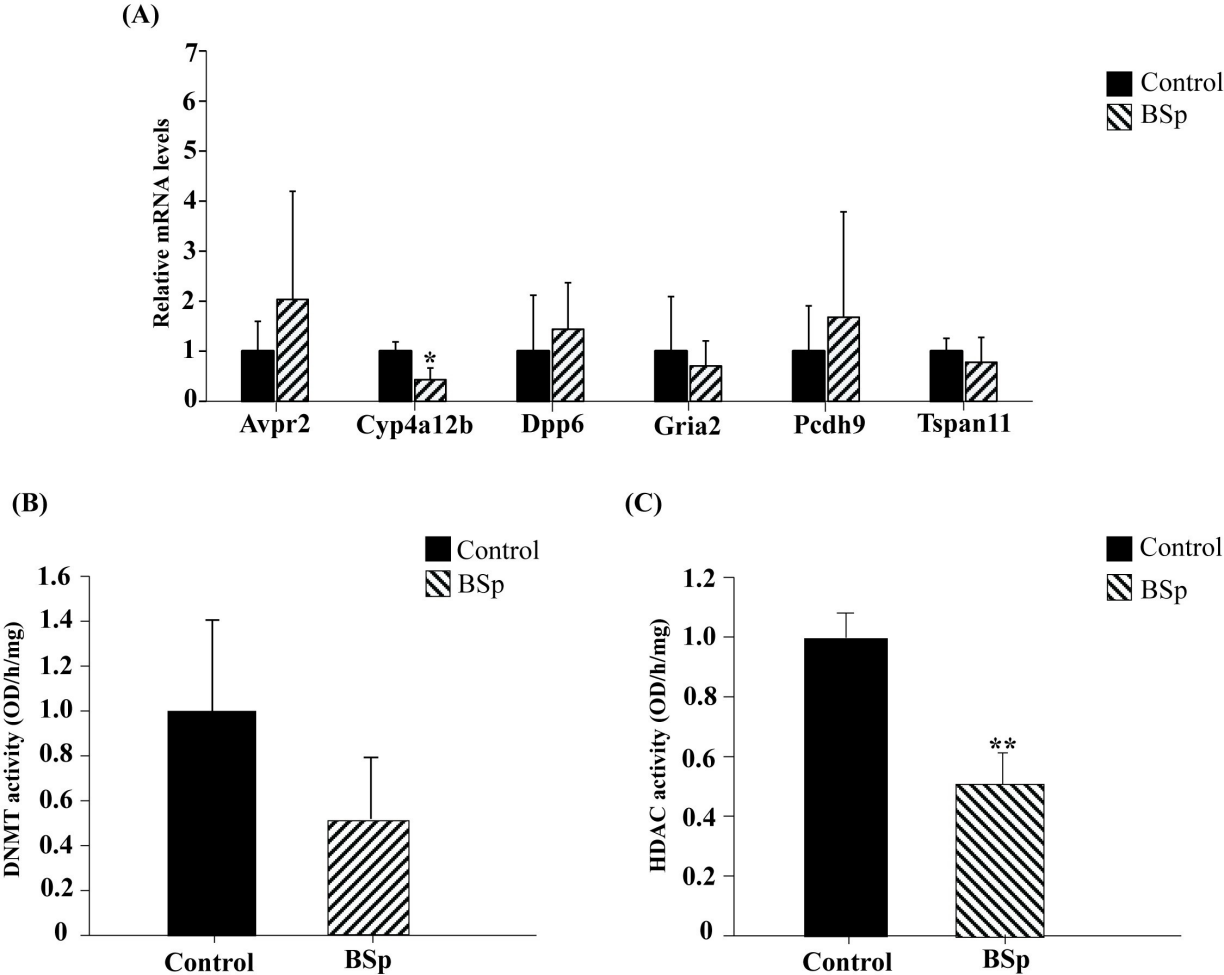
Validation of gene expression changes of identified tumor-related genes and epigenetic enzymatic activity changes in mammary tumors of the offspring mice by maternal BSp treatment. **(A)** Validation of the identified target gene expression such as *Avpr2*, *Cyp4a12b*, *Dpp6*, *Gria2*, *Pcdh9* and *Tspan11* in mammary tumors of the offspring mice using qRT-PCR. The experiments were performed in triplicate from three independent experiments and further normalized to internal control and calibrated to levels in control samples. Columns mean, bars, standard error. *p value < 0.05. **(B)** DNMT activity and **(C)** HDAC activity in SV40 offspring mice by maternal dietary administration of Control or BSp treatment.

To further validate the identified target genes that showed significant changes in DE and DM patterns in response to maternal BSp treatment, we evaluated gene expression of *Avpr2*, *Cyp4a12b*, *Dpp6*, *Gria2*, *Pcdh9* and *Tspan11* genes in the offspring mammary tumors using qRT-PCR. We found that maternal BSp treatment induced mRNA expression changes in all tested genes as shown in Fig 6A. Studies have revealed that amongst the 6 identified target genes, *Dpp6* [39] and *Pcdh9* [40] were Tumor suppressor genes (TSGs) and *Avpr2* [41], *Cyp4a12b* [42], *Dpp6* [39], *Gria2* [43], *Pcdh9* [40] and *Tspan11* [44] were key tumor-related transcripts that can regulate various pathways during carcinogenesis (Table 3). Consistently, our results showed that *Avpr2*, that was upregulated and hypomethylated in genome-wide analysis, was also upregulated validated by qRT-PCR (*Avpr2* FC_BSp_  =  2.04). Subsequently, the results of qRT-PCR in *Cyp4a12b*, *Gria2* and *Tspan11* genes also displayed down-regulated expression patterns in offspring tumors with maternal BSp treatment (*Cyp4a12b* FC_BSp_  =  0.43*, *Gria2* FC_BSp_  =  0.71 and *Tspan11* FC_BSp_  =  0.78). These results indicate that maternal dietary administration of BSp could influence these key gene expression and epigenetic changes, which may partially contribute to its preventive effects in inhibiting early BC in SV40 offspring mice. Table 4 summarizes the functions of the identified target genes, the changes at transcriptional and methylation levels in genome-wide level and qRT-PCR results.

**Table 4:** Summary of identified key genes in response to maternal BSp treatment.

Gene	Epigenetic regulations and function in cancer	RNA-Seq	Realtime RT-PCR	RRBS	References
*Avpr2*	A hypomethylated transcript enriched in neuroactive ligand receptor interaction	DEG, Significant increase (4.26 fold)	Relative expression increase (2.04 fold)	Hypomethylation	[45]
*Cyp4a12b*	A key transcript that actively participates in the metabolism of various carcinogens	DEG, Significant decrease (0.13 fold)	Significant decrease (0.43 fold)	Hypermethylation	[46]
*Dpp6*	A novel target tumor suppressor gene (TSG) for epigenetic regulation such as DNA methylation (Dnmt3b activity)	DEG, Significant decrease (0.19 fold)	Relative expression increase (1.44 fold)	Hypermethylation	[47]
*Gria2*	Changes in DNA methylation and histone modifications with hypermethylated CpG sites	DEG, Significant decrease (0.14 fold)	Relative expression decrease (0.71 fold)	Hypermethylation	[43]
*Pcdh9*	Tumor suppressing gene with Aberrant DNA hypermethylation of Pcdh9 induced tumor cell arrest at G0/G1 phase	DEG, Significant decrease (0.23 fold)	Relative expression increase (1.68 fold)	Hypermethylation	[48]
*Tspan11*	A novel invasion-related gene as a tumor prognostic indicator associated with poor prognosis	DEG, Significant decrease (0.12 fold)	Relative expression decrease (0.78 fold)	Hypermethylation	[44]

### Dietary treatment with BSp influenced global epigenetic profiles

To determine maternal fed BSp impacts on global epigenetic profiles, we performed ELISA-based experiments to assess global epigenetic enzymatic changes in DNMTs and HDACs activity. We found a relative decline in enzymatic activity of DNMTs (Fig 6B) among Control and BSp treatment group. Additionally, we observed a significant decrease in HDAC enzymatic activity (Fig 6C). Our results indicate that dietary administration of BSp may affect DNA methylation and histone modification through influencing the relevant epigenetic enzymatic activities in SV40 offspring mice.

## Discussion

Various studies and clinical trials have demonstrated that cruciferous vegetables and their phytochemical extracts such as SFN and indole-3-carbinol (I3C) are efficient therapeutic and chemopreventive agents [20,21,23,25,49]. Consumption of cruciferous vegetables, as exemplified by the BSp diet, is regarded as a "epigenetics diet" that is capable of modulating epigenetic pathways and reversing aberrant epigenetic markers leading to cancer prevention and treatment effects [49]. SFN, the most prevalent and bioactive ingredient in the BSp diet, has been found to be an effective HDAC inhibitor that specifically targets histone acetylation processes [26]. Previous studies have suggested that the preventive and therapeutic effects of BSp may elicit through multiple mechanisms such as regulation of apoptosis, cell cycle arrest and further targeting PTEN by regulation of the MYC-WWP1 inhibitory pathway [50–52]. In our previous research, we discovered that BSp is a potent preventive and therapeutic agent against BC *in vitro* and *in vivo* [53,54]. Our recent studies also indicate that the temporal impacts of early-life BSp administration result in differences in susceptibility to breast cancer later in life [11,27]. Although many studies on cruciferous vegetable-derived drugs have revealed efficacy, safety, pharmacokinetics, and molecular processes, there are still many open questions that remain unanswered such as the impact of this dietary botanical on transcriptomic and methylomic profiling changes.

Therefore, in this study, we extended our previous findings by evaluating the effects of maternal feeding of BSp in the offspring of BC transgenic mouse model, SV40, and implementing genome-wide mechanistic exploration via multi-omics study. As a result, multi-omics approach allows incorporation of transcriptome and methylome data to better understand how epigenetic mechanisms influence gene transcriptional profiling changes in genome-wide perspective due to maternal administration of bioactive BSp diet. Overall, this integrative analysis of multi-omics data could eventually contribute towards systematically and holistically understanding the impact of maternal nutrition on a complex crosstalk between the epigenome and the transcriptome.

To better comprehend the underlying mechanisms behind the impacts of maternal dietary intervention on mammary cancer prevention or delay, we characterized the global DNA methylation and gene expression patterns in offspring mammary tumors. Our results showed that maternal exposure to BSp diet resulted in genome-wide DNA methylation and transcriptomic profile changes in several critical transcripts. We detected numerous target genes with a strong correlation between gene expression and DNA methylation changes using integrated analysis (Table 4).

For example, maternal BSp treatment significantly upregulated gene expression of *Avpr2*, which is known to play an essential role in the regulation of salt and water homeostasis in kidney. A study reported that *Avpr2* was associated with G-protein-couple-receptor signaling pathway wherein the transcript was found to be upregulated 2-fold in squamous cell carcinoma causing overexpression of miR-10b profile in head and neck cancer [55]. The results in our study also thereby identified *Avpr2* as a key transcript which was upregulated and hypomethylated in offspring breast tumors with maternal BSp treatment. Additionally, our analyses also revealed that maternal feeding of BSp resulted in down-regulated and hypermethylated changes in several transcripts such as *Cyp4a12b*, *Gria2* and *Tspan11*. The *Cyp4a* subfamily of cytochrome P450 enzymes is often found in the liver, kidney, intestine, lung, heart, and brain of mammals such as rats, rabbits, mice, and humans [42]. Another study in hepatic transcriptomic profiling indicates that *Cyp4a12b* gene was down-regulated and associated with low expression metabolic pathways [56]. There are conflicting reports of *Gria2* gene function in cancer biology. For instance, *Gria2* expression was found to increase in uterine leiomyoma and gastrointestinal neuroendocrine carcinoma in comparison to normal tissues [57–59]. In disparity, *Gria2* expression was down-regulated in high-grade glioma inhibiting thereby tumor cell proliferation and further inducing apoptosis [60]. An investigation in colon adenocarcinoma revealed that down-regulation of *Tspan11 gene* was positively correlated with infiltration of CD_4_+ T cells, macrophages, neutrophils and dendritic cells [44]. Similar to *Gria2*, there have also been contradictory findings for TSGs such as Dipeptidyl peptidase (*Dpp*) family genes and protocadherin 9 (*Pcdh9)* genes. A study reported that *Dpp3* and *Dpp9* mRNA expression levels were considerably elevated in BC tissues, whereas *Dpp4*, *Dpp6*, and *Dpp8* mRNA expression levels were down-regulated in BC tissues compared to normal breast tissues [61]. An investigation reported hypermethylation of *Pcdh9* (TSG) can induce cell cycle arrest at G0/G1 phase in hepatocellular carcinoma [48]. In our analyses, we identified *Cyp4a12b*, *Gria2*, *Tspan11 Dpp6* and *Pcdh9* genes were down-regulated and hypermethylated, suggesting these gene expression may be regulated by epigenetic mechanisms in response to maternal BSp treatment.

In the current study, we focused on exploring the potential epigenetics mechanisms of maternal feeding of BSp on BC development because SFN, the bioactive compound enriched in BSp, has been well demonstrated due to its epigenetic modulatory effects. Our research provided important evidence indicating that maternal intake of SFN-enriched BSp diet, a beneficial epigenetics diet, can induce genome-wide DNA methylation and subsequent transcriptomic profiles changes in the offspring, contributing to its chemopreventive effects on breast cancer later in life. Overall, our results revealed that maternal administration of BSp dietary botanicals can prevent mammary cancer development in the offspring through regulation of gene expression and DNA methylation levels based on an unbiased examination of DE and DM genes as well as functional characterization. Our findings suggest that maternal BSp consumption can influence epigenetic and transcriptional profiles in key tumor-related genes, resulting in early-life preventive effects such as slowing or delaying breast carcinogenesis in the offspring. These discoveries may contribute to a better understanding of maternal dietary intervention in prevention of BC in a mechanistic perspective.

## Supporting information

S1 Checklist A checklist of full ARRIVE 2.0 guidelines. (PDF)

S1 Fig Boxplot for sample across Control and BSp treatment group. Boxplot of distribution of transcript counts after normalization in Control (N  =  3) and the BSp (N  =  4) treatment group.
(TIF)

S2 Fig Normalized histogram of RNA-Seq log 2 (fold change) values. Histogram demonstrating normal distribution of total feature by counts across different samples in Control (N  =  3) and the BSp (N  =  4) treatment group wherein y-axis represents the total frequency across 7 samples and x- axis represents overall sample counts.
(TIF)

S3 Fig Three-dimensional Principal component analysis (PCoA) plot across different treatment groups. Three-dimensional scatter plot of the first three principal component (PC) of the data. Each point represents an RNA-Seq sample in Control (N  =  3) and the BSp (N  =  4) wherein samples with similar gene expression are clustered together.
(TIF)

S1 File Reference list of transcripts with P-value and fold change in BSp treatment group. (XLSX)

S2 File A list of differentially expressed (DE) genes in BSp treatment (p<0.05) and calculated fold change at transcriptome level. (XLSX)

S3 File A comprehensive list of differentially methylated loci (DML) in Control and BSp treatment group at methylome level. (XLSX)

S4 File A comprehensive list of differentially methylated regions (DMRs) in Control and BSp treatment group at methylome level. (XLSX)

S5 File A comprehensive list of differentially methylated (DM genes in BSp treatment (p<0.05) and calculated fold change at transcriptome level. (XLSX)

## Abbreviations

AD: Ad Libitum
AML: Acute myeloid leukemia
Avpr2: Arginine vasopressin receptor 2
BC: Breast cancer
BSp: Broccoli sprouts
CYP: Cytochrome P450s
Cyp4a12b: Cytochrome P450 4A12B precursor
DE: Differentially expressed
DEGs: Differentially expressed genes
DM: Differentially methylated
DMGs: Differentially methylated genes
DMLs: Differentially methylated loci
DMRs: Differentially Methylated regions
DNA: Deoxyribonucleic acid
DNMTs: DNA methyltransferases
Dpp6: Dipeptidyl peptidase 6
ERK: Extracellular signal regulated kinase
FDR: False-discovery rate
FC: Fold change
GATA3: GATA binding protein 3
Gria2: Glutamate ionotropic receptor AMPA type subunit 2
GO: Gene Ontology
HCC: Hepatocellular carcinoma
HDACs: Histone deacetylases
hTERT: Human telomerase reverse transcriptase
Mylk: Myosin light chain kinase
Myl9: Myosin light chain 9
NSCLC: Non-small cell lung adenocarcinoma
Pcdh9: Protocadherin 9
PCoA: Principal component analysis
PD: Postnatal Days
PTEN: Phosphatase and tensin homolog
qRT-PCR: Quantitative real-time PCR
PI3K: Phosphoinositide 3-kinase
RNA: Ribonucleic acid
RRBS: Reduced representation bisulfite sequencing
SAM: S-adenosyl-L-methionine
SCC: Squamous cell carcinoma
SFN: Sulforaphane
STAT 3: Signal transducer and activator of transcription 3
Tspan11: Tetraspanin 11
WebGestalt: WEB-based Gene SeT AnaLysis Toolkit
5-FU: 5-Fluorouracil

## References

[1] Seyfried TN, Huysentruyt LC. On the origin of cancer metastasis. Critical Reviews™ in Oncogenesis. 2013;18(1–2). doi: 10.1615/critrevoncog.v18.i1-2.40 23237552 PMC3597235

[2] Bray F, Ferlay J, Soerjomataram I, Siegel RL, Torre LA, Jemal A. Global cancer statistics 2018: GLOBOCAN estimates of incidence and mortality worldwide for 36 cancers in 185 countries. CA: a cancer journal for clinicians. 2018;68(6):394–424. 30207593 10.3322/caac.21492

[3] Siegel RL, Miller KD, Jemal A. Cancer statistics, 2016. CA: a cancer journal for clinicians. 2016;66(1):7–30. 26742998 10.3322/caac.21332

[4] King M-C, Marks JH, Mandell JB. Breast and ovarian cancer risks due to inherited mutations in BRCA1 and BRCA2. Science. 2003;302(5645):643–6. doi: 10.1126/science.1088759 14576434

[5] Sadikovic B, Al-Romaih K, Squire J, Zielenska M. Cause and consequences of genetic and epigenetic alterations in human cancer. Current genomics. 2008;9(6):394–408. doi: 10.2174/138920208785699580 19506729 PMC2691666

[6] Hilakivi-Clarke L, Clarke R, Lippman M. The influence of maternal diet on breast cancer risk among female offspring. Nutrition. 1999;15(5):392–401. doi: 10.1016/s0899-9007(99)00029-5 10355854

[7] Meeran SM, Ahmed A, Tollefsbol TO. Epigenetic targets of bioactive dietary components for cancer prevention and therapy. Clinical epigenetics. 2010;1(3):101–16. doi: 10.1007/s13148-010-0011-5 21258631 PMC3024548

[8] Li E. Chromatin modification and epigenetic reprogramming in mammalian development. Nature Reviews Genetics. 2002;3(9):662–73. doi: 10.1038/nrg887 12209141

[9] Heard E, Martienssen RA. Transgenerational epigenetic inheritance: myths and mechanisms. Cell. 2014;157(1):95–109. doi: 10.1016/j.cell.2014.02.045 24679529 PMC4020004

[10] Li S, Chen M, Li Y, Tollefsbol TO. Prenatal epigenetics diets play protective roles against environmental pollution. Clinical epigenetics. 2019;11(1):1–31. doi: 10.1186/s13148-018-0606-9 31097039 PMC6524340

[11] Li Y, Buckhaults P, Li S, Tollefsbol T. Temporal efficacy of a sulforaphane-based broccoli sprout diet in prevention of breast cancer through modulation of epigenetic mechanisms. Cancer Prevention Research. 2018;11(8):451–64. doi: 10.1158/1940-6207.CAPR-17-0423 29764806 PMC6072582

[12] Li Y, Saldanha SN, Tollefsbol TO. Impact of epigenetic dietary compounds on transgenerational prevention of human diseases. The AAPS journal. 2014;16(1):27–36. doi: 10.1208/s12248-013-9538-7 24114450 PMC3877417

[13] Robillard JE, Segar JL. Influence of early life events on health and diseases. Transactions of the american clinical and climatological association. 2006;117:313. 18528483 PMC1500933

[14] Berdasco M, Esteller M. Aberrant epigenetic landscape in cancer: how cellular identity goes awry. Developmental cell. 2010;19(5):698–711. doi: 10.1016/j.devcel.2010.10.005 21074720

[15] Esteller M. Aberrant DNA methylation as a cancer-inducing mechanism. Annu Rev Pharmacol Toxicol. 2005;45:629–56. doi: 10.1146/annurev.pharmtox.45.120403.095832 15822191

[16] Zaidi SK, Van Wijnen AJ, Lian JB, Stein JL, Stein GS. Targeting deregulated epigenetic control in cancer. Journal of cellular physiology. 2013;228(11):2103–8. doi: 10.1002/jcp.24387 23589100 PMC3729622

[17] Skinner MK, Manikkam M, Guerrero-Bosagna C. Epigenetic transgenerational actions of environmental factors in disease etiology. Trends in Endocrinology & Metabolism. 2010;21(4):214–22. doi: 10.1016/j.tem.2009.12.007 20074974 PMC2848884

[18] Dolinoy DC, Weidman JR, Waterland RA, Jirtle RL. Maternal genistein alters coat color and protects Avy mouse offspring from obesity by modifying the fetal epigenome. Environmental health perspectives. 2006;114(4):567–72. doi: 10.1289/ehp.8700 16581547 PMC1440782

[19] Wolff GL, Kodell RL, Moore SR, Cooney CA. Maternal epigenetics and methyl supplements affect agouti gene expression in Avy/a mice. The FASEB Journal. 1998;12(11):949–57. 9707167

[20] Li Y, Meeran SM, Tollefsbol TO. Combinatorial bioactive botanicals re-sensitize tamoxifen treatment in ER-negative breast cancer via epigenetic reactivation of ERα expression. Scientific reports. 2017;7(1):1–15. doi: 10.1038/s41598-016-0028-x 28839265 PMC5570897

[21] Meeran SM, Patel SN, Tollefsbol TO. Sulforaphane causes epigenetic repression of hTERT expression in human breast cancer cell lines. PloS one. 2010;5(7):e11457. doi: 10.1371/journal.pone.0011457 20625516 PMC2897894

[22] Cheung KL, Kong A-N. Molecular targets of dietary phenethyl isothiocyanate and sulforaphane for cancer chemoprevention. The AAPS journal. 2010;12(1):87–97. doi: 10.1208/s12248-009-9162-8 20013083 PMC2811646

[23] Higdon JV, Delage B, Williams DE, Dashwood RH. Cruciferous vegetables and human cancer risk: epidemiologic evidence and mechanistic basis. Pharmacological research. 2007;55(3):224–36. doi: 10.1016/j.phrs.2007.01.009 17317210 PMC2737735

[24] Pledgie-Tracy A, Sobolewski MD, Davidson NE. Sulforaphane induces cell type–specific apoptosis in human breast cancer cell lines. Molecular cancer therapeutics. 2007;6(3):1013–21. doi: 10.1158/1535-7163.MCT-06-0494 17339367

[25] Li Y, Buckhaults P, Cui X, Tollefsbol TO. Combinatorial epigenetic mechanisms and efficacy of early breast cancer inhibition by nutritive botanicals. Epigenomics. 2016;8(8):1019–37. doi: 10.2217/epi-2016-0024 27478970 PMC5066124

[26] Ho E, Clarke JD, Dashwood RH. Dietary sulforaphane, a histone deacetylase inhibitor for cancer prevention. The Journal of nutrition. 2009;139(12):2393–6. doi: 10.3945/jn.109.113332 19812222 PMC2777483

[27] Li S, Chen M, Wu H, Li Y, Tollefsbol TO. Maternal epigenetic regulation contributes to prevention of estrogen receptor–negative mammary cancer with broccoli sprout consumption. Cancer Prevention Research. 2020;13(5):449–62. doi: 10.1158/1940-6207.CAPR-19-0491 32184225 PMC7203003

[28] Sharpless NE, DePinho RA. The mighty mouse: genetically engineered mouse models in cancer drug development. Nature reviews Drug discovery. 2006;5(9):741–54. doi: 10.1038/nrd2110 16915232

[29] Bray NL, Pimentel H, Melsted P, Pachter L. Near-optimal probabilistic RNA-seq quantification. Nature biotechnology. 2016;34(5):525–7. doi: 10.1038/nbt.3519 27043002

[30] Love MI, Soneson C, Robinson MD. Importing transcript abundance datasets with tximport. Dim Txi Inf Rep Sample1. 2017;1:5.

[31] Gu H, Smith ZD, Bock C, Boyle P, Gnirke A, Meissner A. Preparation of reduced representation bisulfite sequencing libraries for genome-scale DNA methylation profiling. Nature protocols. 2011;6(4):468–81. doi: 10.1038/nprot.2010.190 21412275

[32] Krueger F, Andrews SR. Bismark: a flexible aligner and methylation caller for Bisulfite-Seq applications. bioinformatics. 2011;27(11):1571–2. doi: 10.1093/bioinformatics/btr167 21493656 PMC3102221

[33] Akalin A, Kormaksson M, Li S, Garrett-Bakelman FE, Figueroa ME, Melnick A, et al. methylKit: a comprehensive R package for the analysis of genome-wide DNA methylation profiles. Genome biology. 2012;13(10):1–9. doi: 10.1186/gb-2012-13-10-r87 23034086 PMC3491415

[34] Wu H, Wang C, Wu Z. A new shrinkage estimator for dispersion improves differential expression detection in RNA-seq data. Biostatistics. 2013;14(2):232–43. doi: 10.1093/biostatistics/kxs033 23001152 PMC3590927

[35] Liao Y, Wang J, Jaehnig EJ, Shi Z, Zhang B. WebGestalt 2019: gene set analysis toolkit with revamped UIs and APIs. Nucleic acids research. 2019;47(W1):W199–W205. doi: 10.1093/nar/gkz401 31114916 PMC6602449

[36] Shen G, Khor TO, Hu R, Yu S, Nair S, Ho C-T, et al. Chemoprevention of familial adenomatous polyposis by natural dietary compounds sulforaphane and dibenzoylmethane alone and in combination in ApcMin/+ mouse. Cancer research. 2007;67(20):9937–44. doi: 10.1158/0008-5472.CAN-07-1112 17942926

[37] Lee DD, Leão R, Komosa M, Gallo M, Zhang CH, Lipman T, et al. DNA hypermethylation within TERT promoter upregulates TERT expression in cancer. The Journal of clinical investigation. 2019;129(1):223–9. doi: 10.1172/JCI121303 30358567 PMC6307937

[38] Bussière FI, Michel V, Fernandes J, Costa L, Camilo V, Nigro G, et al. DNA hypermethylation downregulates telomerase reverse transcriptase (TERT) during H. pylori-induced chronic inflammation. Journal of oncology. 2019;2019. doi: 10.1155/2019/5415761 32082377 PMC7012206

[39] Miettinen JJ, Kumari R, Traustadottir GA, Huppunen M-E, Sergeev P, Majumder MM, et al. Aminopeptidase expression in multiple myeloma associates with disease progression and sensitivity to melflufen. Cancers. 2021;13(7):1527. doi: 10.3390/cancers13071527 33810334 PMC8036322

[40] Tayrac Md, Etcheverry A, Aubry M, Saïkali S, Hamlat A, Quillien V, et al. Integrative genome-wide analysis reveals a robust genomic glioblastoma signature associated with copy number driving changes in gene expression. Genes, Chromosomes and Cancer. 2009;48(1):55–68. doi: 10.1002/gcc.20618 18828157

[41] Sinha S, Dwivedi N, Tao S, Jamadar A, Kakade VR, O’Neil M, et al. Targeting the vasopressin type-2 receptor for renal cell carcinoma therapy. Oncogene. 2020;39(6):1231–45. doi: 10.1038/s41388-019-1059-0 31616061 PMC7007354

[42] Hoch U, de Montellano PRO. Covalently linked heme in cytochrome p4504a fatty acid hydroxylases. Journal of Biological Chemistry. 2001;276(14):11339–46. doi: 10.1074/jbc.M009969200 11139583

[43] Choi C, Choi J, Park Y, Lee Y, Song S, Sung C, et al. Identification of differentially expressed genes according to chemosensitivity in advanced ovarian serous adenocarcinomas: expression of GRIA2 predicts better survival. British journal of cancer. 2012;107(1):91–9. doi: 10.1038/bjc.2012.217 22644307 PMC3389416

[44] Liu J, Jiang C, Xu C, Wang D, Shen Y, Liu Y, et al. Identification and development of a novel invasion-related gene signature for prognosis prediction in colon adenocarcinoma. Cancer cell international. 2021;21(1):1–20. doi: 10.1186/s12935-020-01646-5 33579281 PMC7881672

[45] Ye C, Tao R, Cao Q, Zhu D, Wang Y, Wang J, et al. Whole-genome DNA methylation and hydroxymethylation profiling for HBV-related hepatocellular carcinoma. International journal of oncology. 2016;49(2):589–602. doi: 10.3892/ijo.2016.3535 27221337

[46] Eun HS, Cho SY, Lee BS, Seong IO, Kim KH. Profiling cytochrome P450 family 4 gene expression in human hepatocellular carcinoma. Molecular medicine reports. 2018;18(6):4865–76. doi: 10.3892/mmr.2018.9526 30280198 PMC6236316

[47] Sheikh MA, Malik YS, Yu H, Lai M, Wang X, Zhu X. Epigenetic regulation of Dpp6 expression by Dnmt3b and its novel role in the inhibition of RA induced neuronal differentiation of P19 cells. PLoS One. 2013;8(2):e55826. doi: 10.1371/journal.pone.0055826 23409053 PMC3567024

[48] Lv J, Zhu P, Zhang X, Zhang L, Chen X, Lu F, et al. PCDH9 acts as a tumor suppressor inducing tumor cell arrest at G0/G1 phase and is frequently methylated in hepatocellular carcinoma. Molecular medicine reports. 2017;16(4):4475–82. doi: 10.3892/mmr.2017.7193 28791409 PMC5647006

[49] Li Y, Tollefsbol TO. Impact on DNA methylation in cancer prevention and therapy by bioactive dietary components. Current medicinal chemistry. 2010;17(20):2141–51. doi: 10.2174/092986710791299966 20423306 PMC2904405

[50] Lee Y-R, Chen M, Lee JD, Zhang J, Lin S-Y, Fu T-M, et al. Reactivation of PTEN tumor suppressor for cancer treatment through inhibition of a MYC-WWP1 inhibitory pathway. Science. 2019;364(6441). doi: 10.1126/science.aau0159 31097636 PMC7081834

[51] Bryant CS, Kumar S, Chamala S, Shah J, Pal J, Haider M, et al. Sulforaphane induces cell cycle arrest by protecting RB-E2F-1 complex in epithelial ovarian cancer cells. Molecular cancer. 2010;9(1):1–9. doi: 10.1186/1476-4598-9-47 20196847 PMC2838815

[52] Chu W-F, Wu D-M, Liu W, Wu L-J, Li D-Z, Xu D-Y, et al. Sulforaphane induces G2–M arrest and apoptosis in high metastasis cell line of salivary gland adenoid cystic carcinoma. Oral oncology. 2009;45(11):998–1004. doi: 10.1016/j.oraloncology.2009.05.641 19589718

[53] Li Y, Zhang T, Korkaya H, Liu S, Lee H-F, Newman B, et al. Sulforaphane, a dietary component of broccoli/broccoli sprouts, inhibits breast cancer stem cells. Clinical Cancer Research. 2010;16(9):2580–90. doi: 10.1158/1078-0432.CCR-09-2937 20388854 PMC2862133

[54] Li Y, Zhang T. Targeting cancer stem cells with sulforaphane, a dietary component from broccoli and broccoli sprouts. Future oncology. 2013;9(8):1097–103. doi: 10.2217/fon.13.108 23902242

[55] Severino P, Brüggemann H, Andreghetto FM, Camps C, Klingbeil MdFG, de Pereira WO, et al. MicroRNA expression profile in head and neck cancer: HOX-cluster embedded microRNA-196a and microRNA-10b dysregulation implicated in cell proliferation. BMC cancer. 2013;13(1):1–15. doi: 10.1186/1471-2407-13-533 24209638 PMC3826519

[56] Chandrashekar DS, Golonka RM, Yeoh BS, Gonzalez DJ, Heikenwälder M, Gerwirtz AT, et al. Fermentable fiber-induced hepatocellular carcinoma in mice recapitulates gene signatures found in human liver cancer. PloS one. 2020;15(6):e0234726. doi: 10.1371/journal.pone.0234726 32559205 PMC7304627

[57] Arslan AA, Gold LI, Mittal K, Suen T-C, Belitskaya-Levy I, Tang M-S, et al. Gene expression studies provide clues to the pathogenesis of uterine leiomyoma: new evidence and a systematic review. Human reproduction. 2005;20(4):852–63. doi: 10.1093/humrep/deh698 15705628

[58] Leja J, Essaghir A, Essand M, Wester K, Tötterman TH, Lloyd R, et al. Novel markers for enterochromaffin cells and gastrointestinal neuroendocrine carcinomas. Modern pathology. 2009;22(2):261–72. doi: 10.1038/modpathol.2008.174 18953328

[59] Tsibris JC, Maas S, Segars JH, Nicosia SV, Enkemann SA, O’Brien WF, et al. New potential regulators of uterine leiomyomata from DNA arrays: the ionotropic glutamate receptor GluR2. Biochemical and biophysical research communications. 2003;312(1):249–54. doi: 10.1016/j.bbrc.2003.09.189 14630051

[60] Beretta F, Bassani S, Binda E, Verpelli C, Bello L, Galli R, et al. The GluR2 subunit inhibits proliferation by inactivating Src-MAPK signalling and induces apoptosis by means of caspase 3/6-dependent activation in glioma cells. European Journal of Neuroscience. 2009;30(1):25–34. doi: 10.1111/j.1460-9568.2009.06804.x 19558602

[61] Choy T-K, Wang C-Y, Phan NN, Khoa Ta HD, Anuraga G, Liu Y-H, et al. Identification of Dipeptidyl Peptidase (DPP) Family Genes in Clinical Breast Cancer Patients via an Integrated Bioinformatics Approach. Diagnostics. 2021;11(7):1204. doi: 10.3390/diagnostics11071204 34359286 PMC8304478

