# Supplementary figures and images for: An integrated analysis of the effects of maternal broccoli sprouts exposure on transcriptome and methylome in prevention of offspring mammary cancer
Source: PLoS One. 2022 Mar 9;17(3):e0264858. doi: 10.1371/journal.pone.0264858 (PMC8906608; doi:10.1371/journal.pone.0264858)

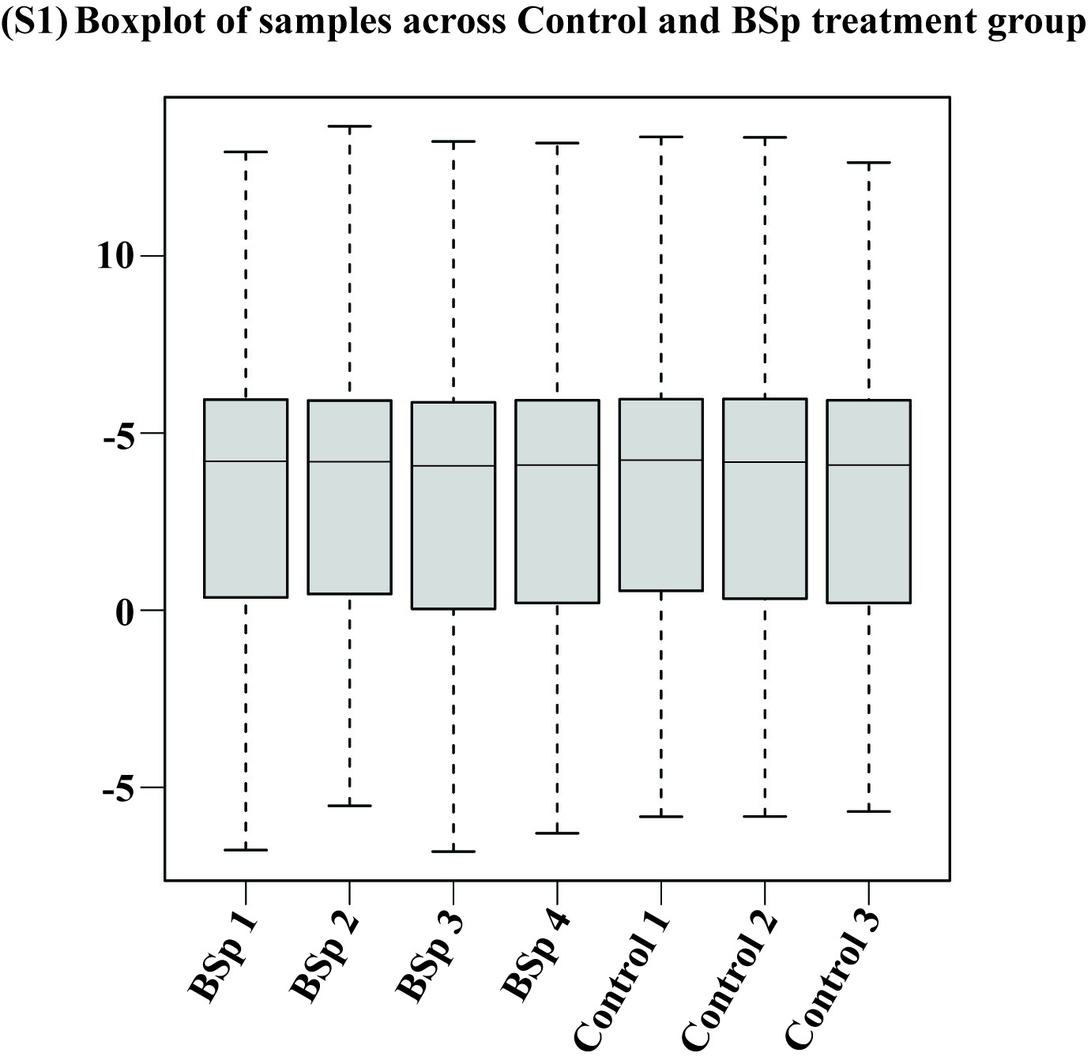

Supplement: S1 Fig — Boxplot of distribution of transcript counts after normalization in Control (N = 3) and the BSp (N = 4) treatment group. (TIF) [file pone.0264858.s002.tif]

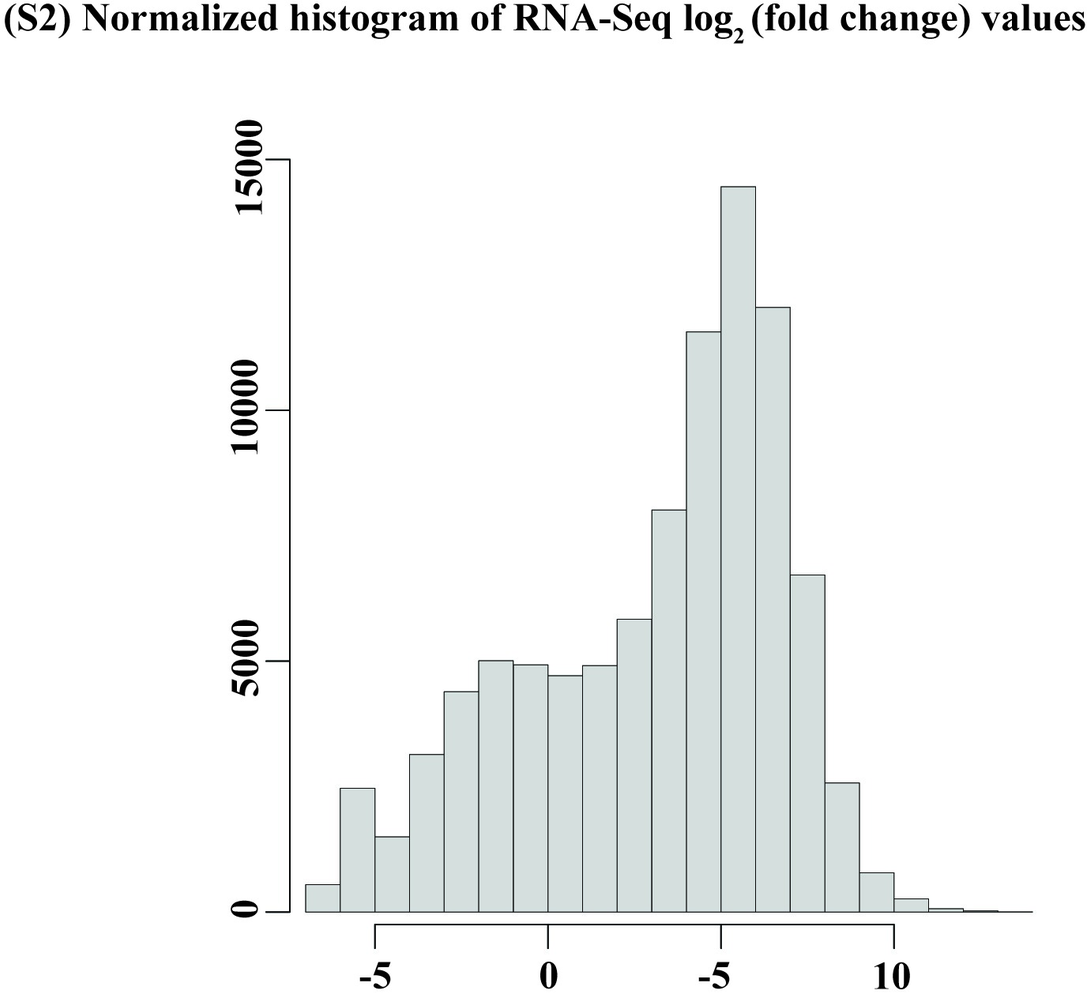

Supplement: S2 Fig — Histogram demonstrating normal distribution of total feature by counts across different samples in Control (N = 3) and the BSp (N = 4) treatment group wherein y-axis represents the total frequency across 7 samples and x- axis represents overall sample counts. (TIF) [file pone.0264858.s003.tif]

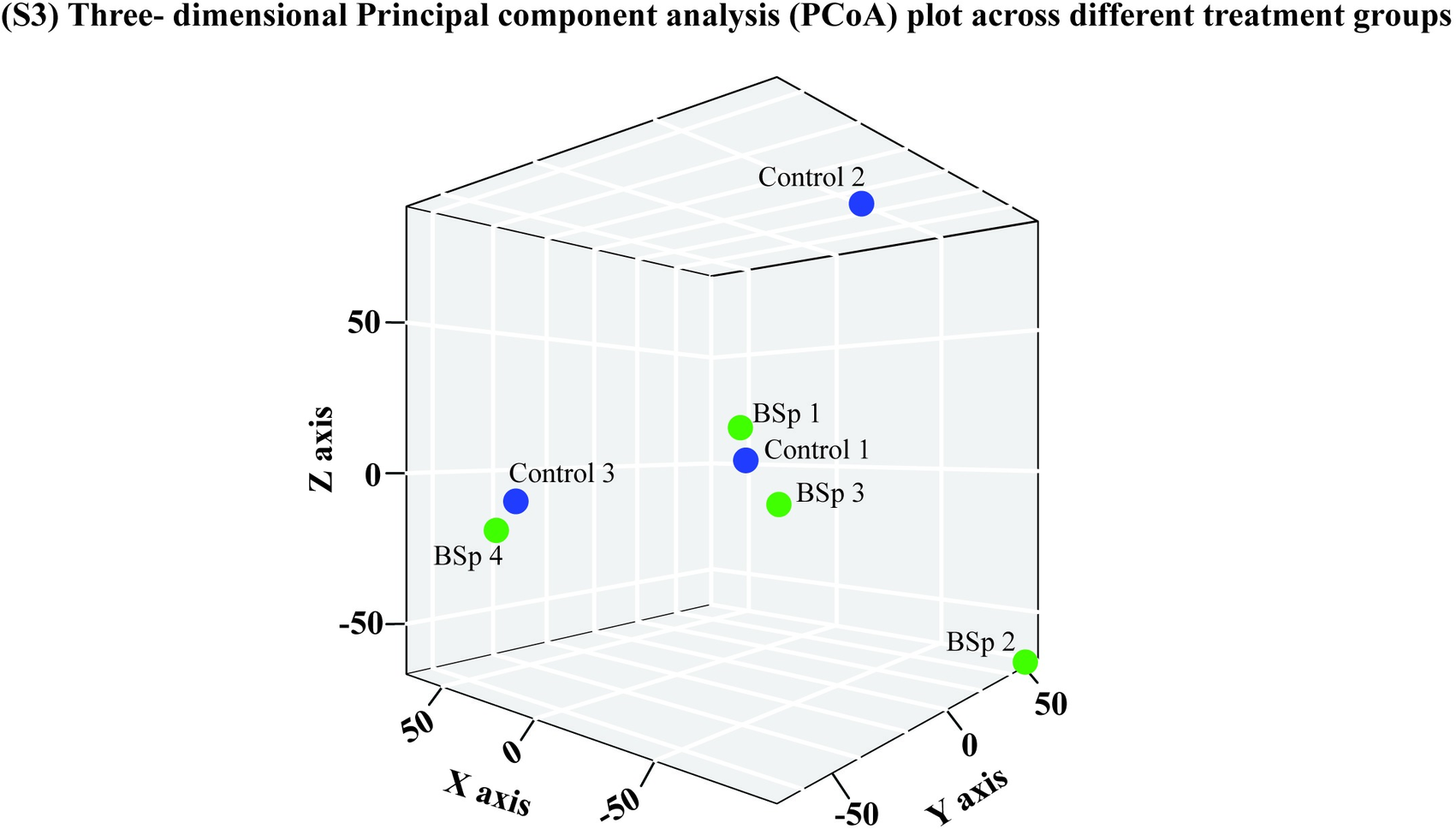

Supplement: S3 Fig — Three-dimensional scatter plot of the first three principal component (PC) of the data. Each point represents an RNA-Seq sample in Control (N = 3) and the BSp (N = 4) wherein samples with similar gene expression are clustered together. (TIF) [file pone.0264858.s004.tif]
